# The impact of pre-transplant ventricular assist device support in pediatric patients with end-stage heart failure on the outcomes of heart transplantation—“a single center experience”

**DOI:** 10.3389/fcvm.2025.1515218

**Published:** 2025-01-23

**Authors:** L. Lily Rosenthal, Carola Grinninger, Sarah Marie Ulrich, Robert Dalla Pozza, Nikolaus A. Haas, Paolo Brenner, Michael Schmoeckel, Sebastian Michel, Christian Hagl, Jürgen Hörer

**Affiliations:** ^1^ Division for Pediatric and Congenital Heart Surgery, Ludwig Maximilian University Munich, Munich, Germany; ^2^ European Pediatric Heart Center Munich (EKHZ), Munich, Germany; ^3^ Department of Congenital and Pediatric Heart Surgery, German Heart Center, University Hospital of the Technical University Munich, Munich, Germany; ^4^ Department of Heart Surgery, Ludwig Maximilian University Munich, Munich, Germany; ^5^ Division of Pediatric Cardiology and Intesive Care, Ludwig Maximilian University Munich, Munich, Germany; ^6^ Munich Heart Alliance (MHA) – German Center for Cardiovascular Research (DZHK), Munich, Germany

**Keywords:** assist device support, pediatric heart failure, cardiomyopathy, pediatric heart disease, pediatric heart transplantation

## Abstract

**Introduction:**

The objective of this study was to examine the impact of ventricular assist device support as a bridge to heart transplantation in children with end-stage heart failure. In light of the limited availability of donor organs, particularly in Europe, the number of children requiring ventricular assist device support is rising at an unavoidable rate.

**Methods:**

We performed a retrospective cohort study of patients who underwent a single and primary pediatric heart transplantation. Patients were divided into two groups: with pre transplant ventricular assist device (VAD) support and without VAD support. The primary outcome was survival at the follow-up evaluation. The time point designated as “time 0” was defined as the time of heart transplantation. Secondary outcome was examined by mean of univariable and multivariable logistic regression, severity of cardiac disease based on ECMO-support pre VAD-support, mean waiting time for transplantation, mean OR time and mean length of hospital stay before and after transplantation.

**Results:**

144 patients could be included in the final analysis. The cumulative survival rate at follow-up period was 67 ± 10% in group 1 vs. 60 ± 6% in group 2 (*P* = 0.769). The mean waiting time (days) on the list was 205 ± 155 in group 1 and 119 ± 69 in group 2 (*P* = 0.002). The mean length of hospital stay (days) was 214 ± 209 in group 1 and 128 ± 91 days in group 2. Early primary-graft-failure was 10% in group 1 and 13% in group 2. Odds ratio [OR] is as follows: 1.992, 95% confidence interval [CI]: 0.983–1.007, *p* = 0.266, aortic clamp time per minutes: OR: 1.008, 95% CI: (0.997–1.019), *p* = 0.164, HLM time per minutes: OR: 0.996, 95% CI: (0.991–1.001), *p* = 0.146, Operation time per minutes: OR: 1.000, 95% CI: (0.995–1.004), *p* = 0.861.

**Conclusion:**

The provision of pre-HTx VAD support does not have an adverse effect on the short- and long-term survival of pediatric patients undergoing HTx. A higher mortality rate was observed among children under three months of age with congenital heart disease. The patients who received VAD support were in a critical condition and required more ECMO support. The results demonstrated a statistically significant correlation between prolonged waiting times and length of hospital stay in group 1. More homogeneous and adequately powered cohorts are needed to better understand the impact of VAD support on posttransplant outcomes.

## Introduction

The use of VAD support as a bridge to heart transplantation (HTx) has demonstrated a consistent upward trajectory over the past decade. A number of factors contribute to the considerably longer waiting periods for pediatric cardiac transplantation in Europe, particularly in Germany, in comparison to the United States. The mean waiting time to date is approximately 1.5 years in Europe, in comparison to approximately three months in the USA (1, 2). As a consequence of the reduced number of available organs for transplantation, there has been an increase in the number of pediatric patients requiring VAD support as a means of providing temporary support prior to a transplant and ensuring survival. A number of studies have demonstrated evidence VAD support as a bridge to HTx exhibit improved renal and liver function, a lower prevalence of malnutrition, and higher survival rates (3–6). Conversely, the use of a VAD is associated with an elevated risk of infection, stroke and pump thrombosis, collectively contributing to an increased morbidity and mortality rate among those awaiting transplantation (7, 8). In this retrospective, single-center cohort analysis, we aimed to analyze the effect of VAD support on heart transplantation—detected posttransplant risk and hazard events—on mortality.

## Methods

Pediatric heart transplantations at our center between October 1988 and July 2024 were retrospectively identified from various data sources. Exclusions criteria for the current analysis were patients aged >18 years and those who died on the waiting list for transplantation, patients with VAD support still awaiting transplantation or who had ventricular recovery and underwent VAD explantation. In addition, those cases of combined organ transplantation (heart and liver, heart and kidney, heart and lung) and re-transplantation. Patients with overt indication for double organ transplantation were refused VAD implantation.

One patient died while receiving ECMO support for severe heart failure in the terminal phase, prior to VAD implantation, and was thus excluded from the dataset. The following data were collected: outcomes of patients in end-stage heart failure on VAD support on the waiting list for transplantation and type of VAD systems used in pediatric patients, recipient's demographics (age, gender, weight, BMI, indication to HTX, pre-VAD/HTx ECMO support, waiting time on the list for transplantation). Donor demographics, including age, gender, weight, BMI, ischemic time, and organ preservation method, were also documented. The perioperative data included the mean ± SD time for skin-to-skin, HLM, aortic cross, ventilation support, ICU, and hospital stay before and after HTX. The 1-year post-transplant data included bleeding, effusion, resternotomy, primary graft failure, re-transplantation, arrhythmia necessitating pacemaker implantation, and neurological complications (cerebral bleeding, cerebral stroke, diaphragm paresis).

The patients were divided into two groups based on their outcomes following transplantation. Group 1 received ventricular assist device (VAD) support, while Group 2 did not receive VAD support. The primary endpoint was mortality at follow-up time, and the secondary endpoint was one-year re-transplant free survival. 1-year post-transplant, the occurrence of bleeding, effusion, resternotomy, primary graft failure, and re-transplantation was identified as indicators of graft failure. The presence of kidney dysfunction was characterized by anuria and ascites and the necessity for dialysis treatment, arrhythmia and the necessity for pacemaker implantation, and neurological complications, including cerebral bleeding, cerebral stroke, and diaphragm paresis. The primary objective of ischemic time was to identify the development of early graft failure.

Secondary outcomes were examined using both univariable and multivariable logistic regression. The objective of the study was the estimation of the impact of ischemic time on the development of early graft failure and the identification of statistically significant differences between the groups of “alive” (defined as survival at follow-up time) and “mortality” (re-transplant free survival defined as death or retransplantation within 1-year post-transplant). The study was reviewed and approved by the local Ethical Committee. (ID: 759-15).

### Statistics

The data is presented in a descriptive manner, with the use of statistics to illustrate the distribution of the data. This includes the mean ± standard deviation for continuous variables, as well as absolute and relative frequencies for categorical variables. The population was divided into two groups, one comprising individuals with pre-HTx VAD and the other without. Subsequently, the aforementioned groups were subjected to comparative analysis employing the requisite statistical tests, namely the *t*-test, Fisher's exact test, and chi-square tests. The threshold for statistical significance was set at *P* < 0.050. Cumulative survival curves were constructed using the Kaplan-Meier method with time 0 defined as the time of HTx. A log-rank test was used to evaluate the difference in survival between the two groups, with the hazard ratio (HR) serving as the metric of comparison. The hazard ratios, confidence intervals, and *p*-values were calculated using logistic regression analysis with 1-year mortality as the dependent variable. The multivariable analysis incorporated risk variables that exhibited a statistically significant association with the dependent variable (*P* ≤ 0.2), as determined by the univariable analysis. The data were analyzed using the IBM SPSS Statistics 29 software program.

### Treatment and follow-up

In order to prevent right ventricular failure, nitric oxide was employed as a standard treatment for many patients in the initial period up to three days following heart transplantation. The immunosuppressive therapy modification was performed in accordance with the standard Stanford protocol (9, 10).

## Results

It is imperative to note that all organ donations were sourced from patients who had been diagnosed with brain death (DBD).

### Outcomes following VAD support

**Figure 1:**
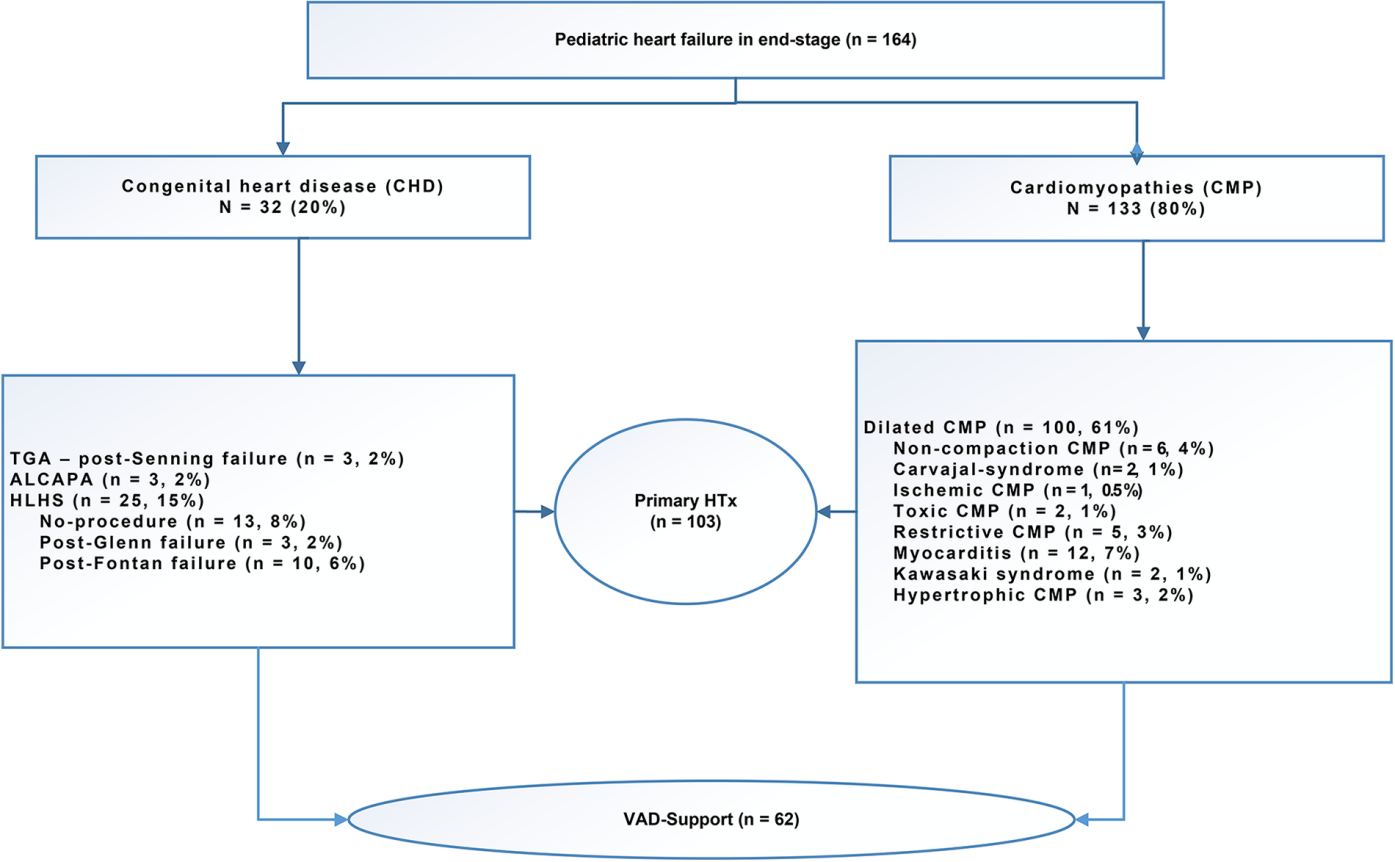
Indication for listing for pediatric heart transplantation with severe end-stage heart failure.

The mean waiting time for transplantation on VAD support was 218 ± 144 days. Following the implantation of ventricular assist devices (VADs) in pediatric patients with congestive heart failure (*n* = 62), these patients were then listed for transplantation. The indication for VAD implantation is invariably the bridge-to-transplant indication. The mean age at the time of VAD implantation was 7 ± 6 years. The VAD support was performed using the Berlin Heart EXCOR (Berlin Heart®, Berlin Heart Inc., The Woodlands, TX, United States) in 41 patients as a LVAD in 34 patients, as a BVAD in 6 patients, and as a RVAD (11) in one patient. The Medos-HIA VAD (Medos®, MEDOS Medizintechnik GmbH, Stollberg, Germany) was used in 10 patients, with 7 receiving the LVAD and 3 receiving the BVAD. Two patients received the Novacor (Novacor®, Baxter Healthcare Corporation, Berkeley, California, USA) as a LVAD. Six patients received a HeartWare® LVAD (HeartWare Inc.), while one patient received a Jarvik® LVAD (Jarvik Heart, INC., 333 West 52nd St., New York, NY 10019, USA), which was among the earliest LVADs developed. Two patients underwent implantation of the HeartMate3 LVAD (Abbott Medical GmbH, Thoratec Corporation; Abbott Park, North Chicago, Illinois, USA) while awaiting a suitable organ (Table 1). Five patients who had undergone VAD implantation demonstrated a recovery of cardiac function and subsequently underwent VAD explantation. The mean duration of VAD support was 212 days (range: 111–484 days). Twelve patients expired while awaiting transplantation, and three patients remained on the waiting list for a suitable organ at the follow-up date of July 31, 2024. The causes of death included cerebral bleeding in six patients and multiorgan failure in another six patients Figure 1.

**Table 1:** Type of mechanical cardiac devices in pediatric patients in end-stage heart failure.

ECMO/ECLS	13 (8%)
Berlin Heart® Excor	41 (26%)
LVAD	34 (21%)
RVAD	6 (4%)
BVAD	1 (1%)
Medos-HIA®	10 (6%)
LVAD	7 (4%)
BVAD	3 (2%)
HeartWare® LVAD	6 (4%)
Novacor® LVAD	2 (1%)
Heart Mate® LVAD	2 (1%)
Jarvik® LVAD	1 (1%)

Values are number (percentage,%).

ECMO, extracorporeal membrane oxygenation; ECLS, extracorporeal life support; LVAD, left ventricular assist device; RVAD, right ventricular assist device; BVAD, biventricular assist device.

### Outcomes following pediatric heart transplantation

#### Recipient's demographic data

**Figure 2:**
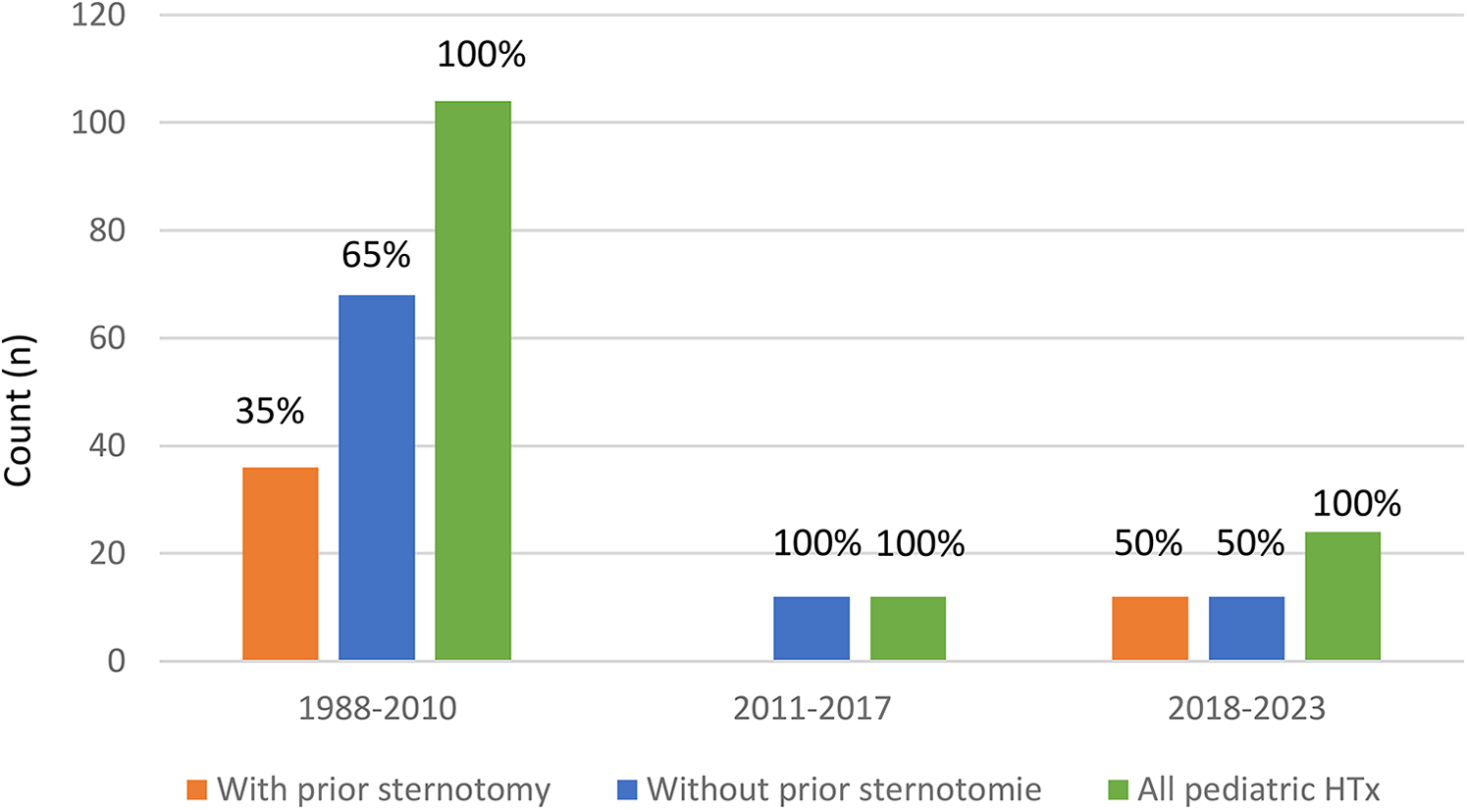
Decreasing number of pediatric heart transplantation dependent on the ears.

The mean age of the recipients at the time of transplantation was 8 ± 6 years for group 1 and 9 ± 7 years for group 2. Group 1 included 19 males, while group 2 had a total of 58 males. The mean weight (kg) was 27 ± 21 in group 1 and 29 ± 22 in group 2. The mean BMI (kg/m^2^) was 17 ± 4 for group 1 and 16 ± 4 for group 2. The indications for transplantation included end-stage CMP in 38 patients in group 1 and 75 patients in group 2, and end-stage congenital heart failure in 3 patients in group 1 and 28 patients in group 2. Prior to VAD support or transplantation, 6 patients in group 1 and 3 patients in group 2 received ECMO/ECLS support. The mean waiting time (days) for transplantation was 205 ± 155 for group 1 and 153 ± 93 for group 2 Table 2, Figure 2.

**Table 2:** Clinical and basic data of recipients.

	Group 1 *N* = 41	Group 2 *N* = 103	All *N* = 144	*p*
Age (y)	8 ± 6	9 ± 7	8.5 ± 6	*0*.*963*
Age groups				*0*.*037*
- <3 months	0	13 (13%)	13 (9%)	
- ≥3 < 6 months	2 (2%)	6 (7%)	8 (6%)	
- ≥6 < 12 months	4 (12%)	4 (3%)	8 (6%)	
- ≥1 < 3 years	8 (19%)	9 (9%)	17 (12%)	
- ≥3 < 6 years	5 (12%)	8 (8%)	13 (9%)	
- ≥6 < 12 years	8 (19%)	15 (15%)	23 (16%)	
- ≥ 12 years	14 (34%)	48 (47%)	62 43%)	
Gender (male)	19 (46%)	58 (56%)	77 (53%)	*0*.*355*
Body weight (per kg)	27 ± 21	29 ± 22	29 ± 22	*0*.*861*
BMI (per kg/m^2^)	16 ± 4	16 ± 4	16 ± 4	*0*.*860*
Indication to HTx				***0***.***012***
CMP	38 (92%)	75 (73%)	113 (78%)	
CHD	3 (3%)	28 (27%)	31 (22%)	
Pre-VAD ECMO (ECLS)- support	6 (15%)	3 (3%)	9 (6%)	***0***.***016***
Waiting time on list (days)	205 ± 155	118 ± 69	153 ± 93	***0***.***002***

Values are mean ± standard deviation or number (percentage,%).

BMI, body mass index; CMP, cardiomyopathy; CHD, congenital heart failure; VAD, ventricular assist device; ECMO, extracorporeal membrane oxygenation; ECLS, extracorporeal life support.

*P* value: The threshold for statistical significance was set at *P* < 0.050.

#### Donor's demographic data

The mean age of the donors was 15 ± 14.5 years for group 1 and 14 ± 13.5 years for group 2. Group 1 included 16 males, while group 2 had a total of 56 males. The mean weight (kg) was 36 ± 25 for group 1 and 36 ± 35.5 for group 2, while the mean BMI (kg/m^2^) was 18 ± 4 for group 1 and 18 ± 5 for group 2. The mean total ischemic time (minutes) was 214 ± 57 for group 1 and 231 ± 45 for group 2. The organ preservation method used in group 1 was HTK in 28 patients, UW2 in 13 patients, and in group 2, HTK in 28 patients, UW2 in 71 patients, and Celsior® in 4 patients Table 3.

**Table 3:** Clinical and basic data of donors.

	Group 1 *N* = 41	Group 2 *N* = 103	All *N* = 144	*p*
Gender (male)	16 (39%)	56 (54%)	72 (50%)	0.139
Age (per year)	15 ± 14.5	14 ± 13.5	15 ± 14	0.710
Body weight (per kg)	38 ± 25	37 ± 26	37 ± 25	0.835
BMI (per kg/m^2^)	19 ± 4	19 ± 5	19 ± 5	0.756
Ischemic time (minutes)	214 ± 58	231 ± 47	227 ± 50	*0*.*068*
DBD	41	103	144	*-*
Organ preservation				*<0*.*001*
HTK	28 (68%)	28 (27%)	56 (39%)	
UW2	13 (32%)	71 (70%)	84 (58%)	
Celsior®	0	4 (4%)	4 (3%)	

Values are mean ± standard deviation or number (percentage,%).

BMI, body mass index; DBD, donation after brain death; HTK, histidine- tryptophan- ketoglutarate solution; UW2, University of Wisconsin solution.

*P* value: The threshold for statistical significance was set at *P* < 0.050.

### Perioperative date

The mean ± standard deviation of the time (in minutes) is as follows: Skin-to-Skin was 363 ± 106 for group 1 and 257 ± 62 for group 2, HLM-time was 185 ± 55 for group 1 and 204 ± 54 for group 2, aortic cross was 78 ± 20 for group 1 and 78 ± 20 for group 2, delayed chest closure was in 11 patients in group 1 and in 32 patients in group 2. The ventilation support duration was 87 ± 51 h in group 1 and 62 ± 43 h in group 2. The ICU duration was 27 ± 22 h in group 1 and 39 ± 25 h in group 2. The hospital stay duration before HTx was 214 ± 209 days in group 1 and 128 ± 91 days in group 2. The duration of hospital stay after HTx was 44 ± 37 days for group 1 and 61 ± 56 days for group 2 Table 4.

**Table 4:** Perioperative data and post-transplant results following pediatric heart transplantation.

	Group 1 *N* = 41	Group 2 *N* = 103	All *N* = 144	*p*
Aortic clamp-time (minutes)	78 ± 21	76 ± 29	76 ± 27	*0*.*697*
Length of ventilation support (hour)	184 ± 142	901 ± 213	768 ± 193	*0*.*599*
ICU length post-transplant (days)	24 ± 23	40 ± 26	36 ± 25	*0*.*759*
Hospital length (days)	211 ± 209	128 ± 90	151 ± 141	***0***.***002***
Post-HTx-ECMO or ECLS	10 (4%)	16 (16%)	26 (17%)	*0*.*237*
Re-HTx at 1-year	0	1 (1%)	1 (1%)	
Graft failure 1-year	2 (5%)	12 (12%)	14 (10%)	*0*.*350*
Bleeding at 1-year	14 (34%)	35 (34%)	49 (34%)	*0*.*681*
Effusion at 1-year	10 (24%)	33 (32%)	43 (30%)	*0*.*562*
Re-sternotomy at 1-year	5 (12%)	18 (17%)	23 (16%)	*1*.*000*
Renal dysfunction at 1-year	9 (22%)	39 (38%)	48 (33%)	*0*.*347*
Dialysis at 1-year	6 (15%)	24 (23%)	34 (21%)	*0*.*831*
Cardiac arrythmia at 1-year	13 (32%)	54 (52%)	67 (46%)	*0*.*097*
Permanent PM-Implantation at 1-year	2 (16%)	7 (7%)	9 (8%)	*0*.*087*
Neurological complication at 1-year	15 (32%)	30 (29%)	45 (30%)	*0*.*560*
*• Cerebral bleeding*	• 5 (11%)	• 11 (11%)	16 (11%)	
*• Ischemic stroke*	• 6 (11%)	• 11 (11%)	17 (11%)	
*• Diaphragm paresis*	• 4 (10%)	• 8 (7%)	12 (8%)	

Values are mean ± standard deviation or number (percentage,%).

ICU, intensive care unit; ECMO, extracorporeal membrane oxygenation; ECLS, extracorporeal life support; PGD, primary graft disorder; HTx, transplantation; PM, pacemaker.

*P* value: The threshold for statistical significance was set at *P* < 0.050.

### Primary endpoint: survival at follow-up time

**Figure 3:**
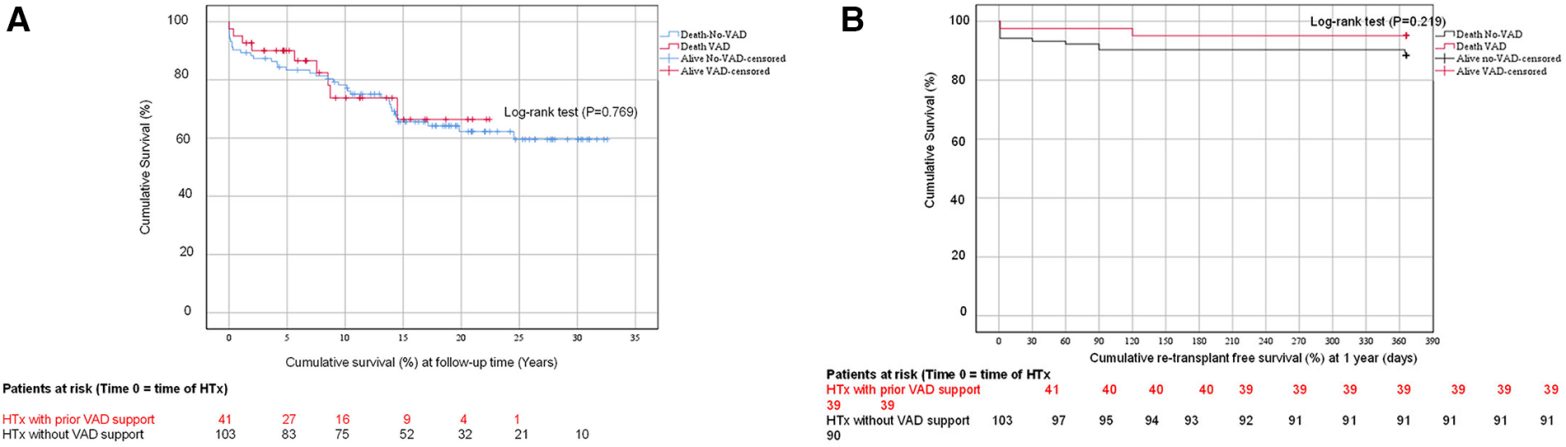
**(A)** The cumulative survival curve (Kaplan-Meier-Curve) of patients with/without ventricular assist device support following HTx. **(B)** The cumulative re-transplant free survival (%) curve (Kaplan-Meier-Curve) of patients with/ without ventricular assist device support at 1-year.

As presented in Figure 3A, the cumulative survival at follow-up time in group 1 was 66 ± 3%, while in group 2, it was 60 ± 6% (*P* = 0.769). At 1-year, 5-year, 10-year, and at follow-up time, the survival rates were 95 ± 4%, 90 ± 5%, 73 ± 8%, and 66 ± 10%, respectively, for group 1. For group 2, the survival rates were 90 ± 3%, 83 ± 4%, and 78 ± 4%, respectively, at 1-year, 5-year, and 10-year follow-up time, and 60 ± 6% at follow-up time.

The mean time to mortality (years) was 5.4 ± 5 for group 1 and 7.5 ± 7 for group 2. The details of age (years), weight (kg), and BMI (kg/m^2^) were 9.3 ± 8.2, 35 ± 30, and 17 ± 5 for group 1, respectively. The *p*-values were 0.970, 0.706, and 0.589, respectively. The crosstabs included gender (male), indication for HTx (CHD vs. CMP), and HLHS observed in group 1: four male patients, eight patients with CMP, and one patient with CHD, and HLHS in none of patients. In contrast, group 2 resulted a total of 19 male patients, 24 patients with CMP, and 12 with CHD. Notably, none of the patients in group 1 or group 2 showed HLHS. Among the patients in group 2, 10 cases of HLHS were observed, with five cases resulting from primary transplantation, two cases from Norwood failure, one case from Glenn failure, and one case from Fontan failure.

### Secondary endpoints

#### 1-year re-transplant free survival

As shown in Figure 3B, the 1-year re-transplant free survival rate was evaluated using a log-rank test (*P* = 0.219). Group 2 observed only one patient aged 17 years who developed early-term post-transplant severe post-graft failure with severe humoral rejection. The patient underwent a re-transplantation after 30 days and is alive. In group 1, two patients (17%) died at 1-year posttransplant. One patient had myocarditis and was 16 years old, and the other had DCM and was 15 years old. In group 2, 10 patients (83%) died at 1-year posttransplant. three (30%) had DCM, with ages ranging from 13 to 17 years, four patients (40%) had HLHS with primary HTx, with ages ranging from 10 to 15 days. One patient (10%) had Glenn failure at the age of 15 years. Another patient (10%) had Shone anomaly at age 4 years. Finally, one patient (10%) had non-compaction cardiomyopathy at age 20 days. The causes of death among patients who succumbed within one year are delineated in the primary endpoint details. The primary cause of death among these patients was identified as primary graft failure and multiorgan failure, particularly in those with HLHS and without VAD support.

The cumulative survival rate at 10-year post-transplant showed a tendency to be higher in patients with CMP (91 ± 5%) than in patients with CHD (78 ± 9%), although this difference was not statistically significant.

### Perioperative data

The mean ischemic time was 194 ± 55 min for group 1 and 209 ± 38 min for group 2. The mean aortic cross-clamp time was 78 ± 21 min for group 1 and 76 ± 29 min for group 2. The mean length of hospital stay was 211 ± 209 days for group 1 and 128 ± 90 days for group 2 (*P* = 0.002). The mean hospital length of stay following transplantation was 44 ± 37 days for group 1 and 61 ± 56 days for group 2 (Table 4).

### Post-transplant results

**Figure 4:**
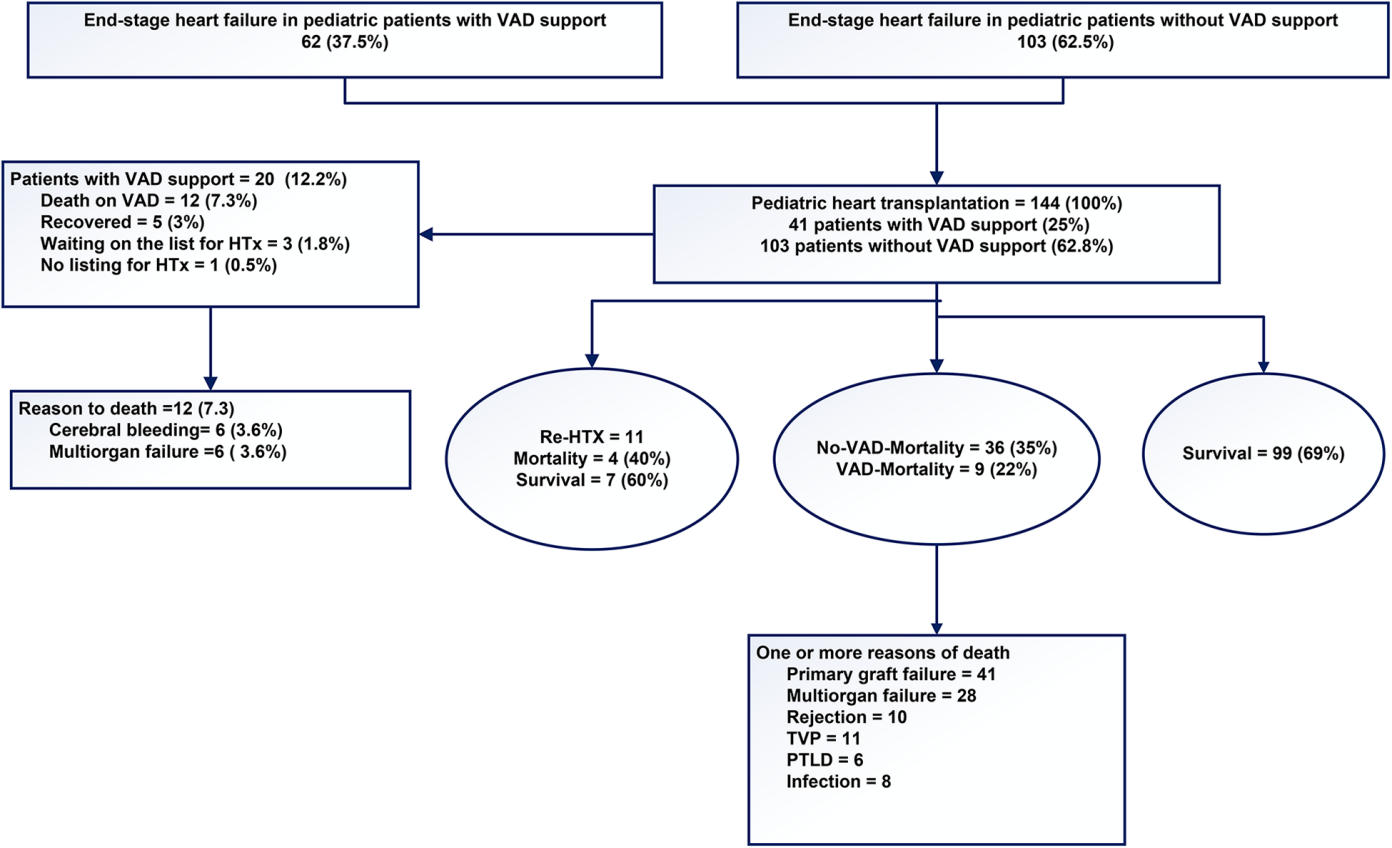
The outcomes of pediatric patients with severe end-stage heart failure.

The outcomes of pediatric HTx with vs. without prior VAD support are presented in Tables 4, 5 and in Figure 4. Early graft failure or dysfunction was observed in 2 (5%) patients in group 1 and in 12 (12%) patients in group 2. Graft dysfunction was observed due to acute rejection and after standard treatment. Recovery of heart function was evidenced by recovery in echocardiography. In Group 2, one patient underwent retransplantation due to severe humoral rejection.1-year posttransplant bleeding, effusion with necessity of resternotomy were in group 1 in 14 (34%), in 10 (24%) and in 5 (12%) and in group 2 were 35 (34%), 33 (32%) and 18 (17%). Renal dysfunction with anuria and ascites with necessity of dialysis for 7–30 days was 9 (22%) in group 1 and 39 (38%) in group 2. Dialysis for 30–90 days was in 6 (15%) in group 1 and 24 (23%) in group 2. Arrhythmias are an expected complication of the procedure; however, they generally convert into sinus rhythm (SR) within 4–8 weeks posttransplant. This result was shown in 13 (32%) patients in group 1 and 54 (52%) patients in group 2. In instances where pacemaker implantation was necessary due to AV block or sinus bradycardia was observed, it was noted in 2 (16%) patients in group 1 and 7 (7%) patients in group 2. It is noteworthy that, in era 1, the majority of patients underwent transplantation using the standard (Shumway) method, which has been associated with an increased risk of arrhythmias.

**Table 5:** Risk factor analysis for 1-year mortality using univariable Cox regression analysis.

	Univariable Cox analysis	
	OR (95% CI)	*p*-value
Recipient's-Age (per years)	1.023 (0.978–1.070)	0.326
Recipient's-Gender	1.118 (0.622–2.009)	0.709
Recipient's-Body weight (per kg)	1.010 (0.997–1.023)	0.132
Recipient's-BMI (per kg/cm^2^)	0.991 (0.855–1.149)	0.909
Indication for HTX	1.304 (0.674–2.521)	0.431
Pre- HTx ECMO/ECLS	1.541 (0.452–5.249)	0.489
Waiting on the list (per days)	0.999 (0.999–1.002)	0.642
Donor's-Age (per years)	0.999 (0.995–1.031)	0.163
Donor's-Gender	0.923 (0.511–1.664)	0.789
Donor's-Body weight (per kg)	1.009 (0.998–1.021)	0.237
Donor's-BMI (kg/cm^2^)	1.072 (1.012–1.135)	0.237
Early/primary graft failure	1.000 (0.113–8.851)	1.000
ECMO/ECLS	1.488 (0.406–5.453)	0.549
Bleeding	0.935 (0.498–1.756)	0.835
Effusion	0.082 (0.572–2.046)	0.809
Resternotomy (because of bleeding)	1.325 (0.632–2.786)	0.457
Renal dysfunction	1.452 (0.786–2.682)	0.234
Dialysis	0.592 (0.131–2.673)	0.495
Cardiac arrhythmia	0.593 (0.317–1.108)	0.101
Pacemaker implantation	1.390 (0.639–3.025)	0.406
Wound infection (no mediastinitis)	0.772 (0.417–1.428)	0.409
Neurological (infarction/bleeding)	0.949 (0.493–1.825)	0.875
Organ preservation solution	6.068 (0.750–49.121)	0.091
Ischemic time per minutes	1.992 (0.983–1.007)	0.266
Aortic clamp time per minutes	1.008 (0.997–1.019)	0.164
HLM time per minutes	0.996 (0.991–1.001)	0.146
OR—time (skin-to-skin) per minutes	1.000 (0.995–1.004)	0.861
Delated chest closure	1.164 (0.626–2.166)	0.631

OR, add ratio; CI, confidence interval; BMS, body mass index; ECMO:, extracorporeal membrane oxygenation; ECLS, extracorporeal life support; HLM, heart lung machine; OR, operating room.

## Discussion

The employment of assist device systems to address acute critical hemodynamic situations has become a standard of care in Europe and many other countries on various continents. A significant proportion of the world's cardiac surgeons, with a notable emphasis on pediatric cardiac surgeons, have attained expertise in this field. Our clinic has been at the forefront of this progress, particularly in the field of pediatric heart transplantation. Notably, our clinic was the second in Germany, after Giessen, to perform the first primary transplant of an infant with HLHS. This study presents a retrospective data analysis of 144 pediatric patients who underwent pediatric heart transplantation at our center over an extended period, from 1988 to 2024.

In recent decades, excellent results have been reported, with low morbidity and rates. There has been a notable shift in the composition of the heart transplantation waiting list, resulting in an extended list of individuals awaiting transplantation for a longer time. As a consequence of prolonged waiting times, children with severe end-stage heart failure require VAD support with greater frequency. During the observed follow-up period, the waiting time on the list increased steadily. A total of 12 patients died while awaiting transplantation, mainly due to multi-organ failure or severe cerebral complications (Figure 4). Recovery of heart function after VAD supporting observed 5 patients (Figure 4). A review of the collective data indicates that the median time spent on the waiting list for pediatric heart transplantation has increased in both groups. As a consequence of the identified factors, the number of heart transplants performed at our center due to the limited availability of donor organs. The waiting period for a compatible heart has increased considerably in Germany and at our center. Even when infants were listed for an ABO incompatible heart transplant (HTx), they often had to wait for extended periods. As a result, this type of transplant becomes unfeasible due to natural antibody formation in all cases from 2015 to the present (11).

The present study reports the results obtained in a small number of pediatric heart transplants by 144 patients with a mean age of 8.5 years. The Kaplan-Meier curve for mortality revealed that the cumulative survival rate at the follow-up time point did not demonstrate a statistically significant difference between the two groups (*P* = 0.769). The analysis of risk factors for one-year mortality showed that univariable Cox regression (OR) revealed that the 95% confidence interval (CI) did not exhibit significant variation across all observed variables. The clinical use of a VAD as a bridge to heart transplantation (HTx) or as a bridge to recovery has been demonstrated to have a favorable impact on survival rates in international reports (3, 5, 12). The reported incidence of age, low weight (BMI), and the etiology of end-stage heart failure have been established as significant risks for morbidity and mortality on outcomes after transplantation (5, 12, 13). We observed for age (per years) OR (95% CI) 1.023 (0.978–1.070, *p* = 0.326), for gender was OR (95% CI): 1.118 (0.622–2.009, *p* = 0.709), for weight (per kg) vs. BMI (per kg/cm^2^) were OR (95% CI): 1.010 (0.997–1.023, *p* = 0.132) and 0.991 (0.855–1.149, *p* = 0.909). Nonetheless, it was observed that the children in group 1 who died within 1-year posttransplant were quite young and small at the time of transplantation. Valuable data and reports presented low weight, low BMI and CHD as indication to heart transplantation as risk factor for mortality (13–16). In the present report, however, the children in Group 1 who died were observed to be quite young and small and they had the indication of CHD for transplantation retrospectively with no significant findings.

A multi-cohort study conducted by Lammers and colleagues demonstrated that extracorporeal life support (ECLS) has a statistically significant impact on mortality (17). Morbidity and mortality rates remain elevated, particularly among patients with congenital heart disease and those who require ECMO prior to VAD implantation (17). ECLS was indicated for the acute treatment of patients who had suffered an acute cardiac arrest or in cases of multiorgan failure. In the event of unsuccessful ventricular function recovery, the patient should be transitioned to a VAD designed for long-term use. The utilization of ECLS did not result in any alterations in either early or late mortality or morbidity, as documented by Groetzner and colleagues in our institution (9). In our results we did not find using ECMO pre VAD vs. pre HTx as a significant risk for mortality at 1-year after transplant, OR (95% CI): 1.541 (0.452–5.249, *p* = 0.489).

In a recent study, Davis and colleagues reported a low incidence of neurological dysfunction in children younger than five years old who received VAD support as a bridge to HTx (18). Morales and colleagues demonstrated that mortality rates were higher in patients who experienced a stroke following pediatric heart transplantation (19). In the present study, the development of neurological complications was observed in each group; however, the observed outcome did not demonstrate a statistically significant OR (95% CI): 0.949 (0.493–1.825, *p* = 0.875).

It has been posited by certain authors that the indication for pediatric heart transplantation as CHD carries a risk of mortality. This risk is particularly pronounced in patients with a single ventricle who have undergone numerous prior surgical palliations. As demonstrated by Conway et al., children weighing less than 10 kg who received VAD support exhibited higher survival rates than those who received ECLS support. The findings indicate that children with CMP demonstrated superior outcomes when placed on the waiting list in comparison to those with CHD (20, 21). The present study revealed no statistically significant differences. Nevertheless, clinical experience points to the importance of multidisciplinary teamwork, with surgeons, anesthesiologists, and intensive care specialists playing key roles in the care of patients with SV. These specialists must possess extensive experience to ensure the appropriate treatment of patients during their intensive care unit stay, a period that is known to be particularly sensitive.

In univariable Cox regression was indication (CHD vs. CMP) OR (95% CI): 1.304 (0.674–2.521, *p* = 0.431). Single-ventricle physiology represents a significant challenge in the surgical and anesthesiologic management of patients with failed single ventricle physiology at the time of VAD implantation or at the time of transplantation (22). In their study, Kanaya et al. demonstrated the long-term efficacy of VAD support in children with CHD. They also found that children with end-stage CHD and VAD support demonstrated a higher incidence of complications than those with CMP (23). It was of significant importance to consider the renal and nutritional status of patients at the time of heart transplantation (HTx). Children who received VAD support while awaiting HTx exhibited superior outcomes with respect to nutritional status and a reduced prevalence of malnutrition at the time of transplantation. Moreover, they demonstrated augmented renal and hepatic functional recuperation, which is presumably attributable to the enhanced hemodynamic performance and diminished risk of chronic kidney disease subsequent to pHTx (3, 4, 24, 25).

The present study was initiated with the transplant procedure. It was imperative to examine the specific functions of the organs with and without VAD support prior to the transplant to ascertain the potential positive influence of VAD on organ function. Post-transplant, renal function was compromised up to one year after transplantation in 9 (22%) subjects in group 1 and 39 (38%) subjects in group 2. However, it is noteworthy that a significant proportion of these patients exhibited a recovery in kidney function by the time they were discharged from the hospital. A total of six (15%) patients in group 1 and 14 (23%) patients in group 2 required dialysis treatment during the one-year observation period. This proportion was especially high in patients with a Fontan failure or Glenn failure as an indication for transplantation, with dialysis being required for over six months in these cases. In the one-year period following transplantation, a Cox regression analysis was employed to examine the renal function mortality rate. The resultant estimate [odds ratio (OR): 1.452, 95% confidence interval (CI): 0.786–2.682, *p* = 0.234] indicated that renal function was not a significant predictor of mortality. A similar analysis was conducted to examine the association between dialysis necessity and mortality, yielding an odds ratio of 0.592 (95% CI: 0.131–2.673, *p* = 0.495), suggesting that dialysis did not significantly increase the risk of mortality. A considerable number of authors have presented evidence that graft failure leads to mortality. The following evidence has been presented by Conway et al. indicate that severe primary graft dysfunction continues to be a significant factor contributing post-transplant morbidity in infant heart transplant recipients, with no evident change in prevalence over the past two decades. In the present study, graft failure was not observed to be statistically significant [OR (95% CI): 1.000 (0.113–8.851), *p* = 1.000]. The analysis of graft dysfunction, as indicated by the use of ECMO/ECLS post-transplantation, also did not reveal a statistically significant association [OR (95% CI): 1.488 (0.406–5.453), *p* = 0.549]. In one patient from group 2, re-transplantation was necessary due to graft failure and severe humoral rejection. Infants who have undergone heart transplantation and who present with severe primary graft dysfunction tend to have a poor prognosis with regard to graft survival. While certain recipient risk factors are not susceptible to modification, the avoidance of the other risk factors may serve to mitigate further risk in infants at high risk of developing severe primary graft dysfunction. In accordance with the findings of other researchers in the field, the utilization of older donor organs has been associated with an increased rate of graft failure in recipients. However, within the context of pediatric heart transplantation, our approach entails the deliberate avoidance of age-mismatch, weight-mismatch, and body-surface-area-mismatch between donor and recipient, to the greatest extent feasible. This was particularly the case if one avoided longer ischemic time of over four hours and donor-to- recipient weight ratio of between ≥0.9 and <2.3 (26). Prolonged total donor ischemic time has been demonstrated to have an adverse effect on the donor organ and the development of primary organ failure (27). In the present risk factor analysis for one-year mortality in ischemic time per minute, the odds ratio [OR] is as follows: 1.992, 95% confidence interval [CI]: 0.983–1.007, *p* = 0.266, aortic clamp time per minutes: OR: 1.008, 95% CI: (0.997–1.019), *p* = 0.164, HLM time per minutes: OR: 0.996, 95% CI: (0.991–1.001), *p* = 0.146, Operation time per minutes: OR: 1.000, 95% CI: (0.995–1.004), *p* = 0.861, organ preservation solution: OR: 6.068, 95% CI: (0.750–49.121), *p* = 0.091, and delayed chest closure: OR: 1.164, 95% CI: (0.626–2.166), *p* = 0.631, with no significant for mortality for any variables.

### Limitations

The study's primary limitations stem from its retrospective design and the relatively limited duration of the follow-up period. The age and indication of each group were found to be heterogeneous. Consequently, a match study could not be conducted due to the heterogeneity of age and indication. The present study linked three major clinical time periods of data contained with decreasing number of transplantation and very long waiting time for transplantation in our center. Nonetheless, it is hypothesized that each investigation within this field in Europe will contribute significant information to the existing corpus of evidence and will also function as a data foundation for potential future analyses. It is noteworthy that guidelines may be rendered more robust if they are grounded in a comprehensive, developing set of evidence.

## Conclusion

The provision of pre-HTx VAD support does not have an adverse effect on the short- and long-term survival of pediatric patients undergoing HTx and enables children with terminal heart failure survival until transplantation in an environment of significant donor/organ shortage. A higher mortality rate was only observed among children under three months of age with congenital heart disease (i.e HLHS and primary transplantation). Based on these results we would encourage other teams to use VAD more often and at an early stage of severe heart failure. More homogeneous and adequately powered cohorts are however needed to better understand the impact of VAD support on posttransplant outcomes.

## Data Availability

The original contributions presented in the study are included in the article/Supplementary Material, further inquiries can be directed to the corresponding author.
